# An Intelligent Gear Fault Diagnosis Methodology Using a Complex Wavelet Enhanced Convolutional Neural Network

**DOI:** 10.3390/ma10070790

**Published:** 2017-07-12

**Authors:** Weifang Sun, Bin Yao, Nianyin Zeng, Binqiang Chen, Yuchao He, Xincheng Cao, Wangpeng He

**Affiliations:** 1 School of Aerospace Engineering, Xiamen University, Xiamen 361005, China; Vincent_suen@126.com (W.S.); yaobin@xmu.edu.cn (B.Y.); zny@xmu.edu.cn (N.Z.); heyuchao1993@126.com (Y.H.); 19920151153746@stu.xmu.edu.cn (X.C.); 2 School of Aerospace Science and Technology, Xidian University, Xi’an 710071, China; hewp@xidian.edu.cn

**Keywords:** intelligent fault diagnosis, dual-tree complex wavelet transform (DTCWT), convolutional neural network (CNN), pattern recognition

## Abstract

As a typical example of large and complex mechanical systems, rotating machinery is prone to diversified sorts of mechanical faults. Among these faults, one of the prominent causes of malfunction is generated in gear transmission chains. Although they can be collected via vibration signals, the fault signatures are always submerged in overwhelming interfering contents. Therefore, identifying the critical fault’s characteristic signal is far from an easy task. In order to improve the recognition accuracy of a fault’s characteristic signal, a novel intelligent fault diagnosis method is presented. In this method, a dual-tree complex wavelet transform (DTCWT) is employed to acquire the multiscale signal’s features. In addition, a convolutional neural network (CNN) approach is utilized to automatically recognise a fault feature from the multiscale signal features. The experiment results of the recognition for gear faults show the feasibility and effectiveness of the proposed method, especially in the gear’s weak fault features.

## 1. Introduction

With the rapid development of modern industry and technology, industrial applications are becoming more complicated and more precise. These changes put forward higher requirements for equipment maintenance. Rotating machinery is an important component in industrial applications, and has been widely used in many crucial areas, but any potential fault may lead to enormous economic loss [1]. Therefore, mechanical condition monitoring and fault diagnosis (CM-FD) for rotating machinery to avoid accidents and increase machine reliability has become an important research area in industry [2].

Known as key elements in rotating machinery, gears are widely used in manufacturing industry and have received significant attention in the field of CM-FD. Typical gear faults include chipped teeth, tooth breakage, root crack, wear, pitting, and surface damage [3]. These failure forms may lead to system imbalance and machining precision deterioration. To diagnose multiple gear faults, fault identification has become an important subject for extensive research for the past few decades [4,5].

**Figure 1:**
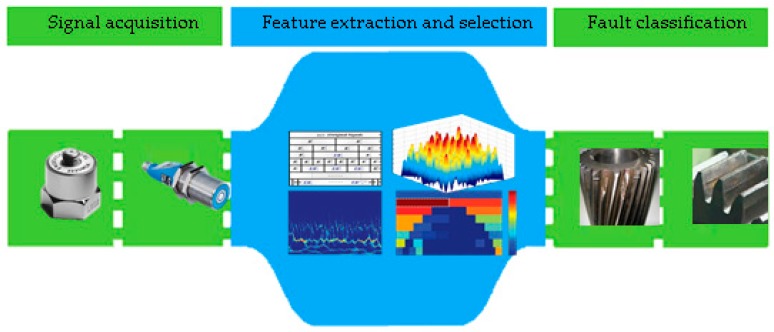
Traditional diagnosis method.

The framework of traditional fault diagnosis includes three main steps: (1) signal acquisition; (2) feature extraction and selection; and (3) fault classification [6], as shown in Figure 1. A proper signal acquisition method is the premise and crux of the effective usage of accurate CM-FD. According to the data acquisition media, CM-FD can be divided into the following categories: vibration [7], oil analysis [8], ultrasonic/acoustic emission [9,10], and infrared monitoring [11]. Vibration signal analysis of mechanical systems is the most common and effective approach for CM-FD [12]. Multifarious diagnostic approaches based on a spectrum analysis of the vibration signal have been proposed, and have achieved huge success [13]. Time domain, frequency domain, and time-frequency domain are the three main methods for signal processing and feature extraction. Time-frequency analysis offers both the time and frequency domain local minutiae characteristics. Wavelet transform utilizes a decaying wave atom, whose translation and scaling form multi-resolution analysis (MRA) [14]. The construction of wavelet bases has attracted extensive attention across the world. The dual tree complex wavelet transform (DTCWT) is a recent enhancement to conventional discrete wavelet transforms because it has the attractive properties of approximate shift-invariance and inhibited frequency aliasing [15]. Chen proposed an iterated method for constructing a dual tree complex wavelet base with the enhanced frequency aliasing property [16]. Wang demonstrated the robustness of DTCWT in extracting vibration features interfered by strong noises [17]. In the above studies, all of the projects needed to speed lots of time in data analyzing according to prior knowledge and experience.

The pattern recognition concept can be defined as identifying or classifying complex signal samples or objects [18]. Therefore, CM-FD can be also considered as a pattern recognition problem. Among various frameworks in pattern recognition, supervised and unsupervised learning are the two major manners. Unsupervised learning mainly focuses on the hidden structure description of unlabeled data. Some literature [19,20] has exhibited the potential possibility to perform CM-FD in a completely unsupervised manner. On the other hand, the supervised manner exhibited an extraordinary ability for the classification problem. The supervised learning manner mainly focuses on the relationship between the input explanatory independent vector of a feature and the dependent class or cluster [21]. N. Saravanan and K.I. Ramachandran [22] proposed a method based on a discrete wavelet transform (DWT) and an artificial neural network (ANN), and an accuracy of 95% was obtained. In [23], the authors engaged Continuous wavelet transform (CWT) and ANN into a fault classification, and the fault estimation was 98.28% accurate. A method in [24] using empirical mode decomposition (EMD) and an ANN was proposed by Ali, J.B. et al., and the classification accuracy result was 93%. Xian [25] proposed a mechanical failure classification using DWT and a Support vector machine (SVM); the classification result of failure in validation was 94.33%. In [26], the authors presented a feature extraction method, and the average classification result was reported as 95.76%. A method [27] using EMD and SVM was proposed by Babu, N.R. and Mohan, B.J., and the fault classification accuracy result was 95.33%. In this research, supervised classification is employed for the gear fault diagnosis. 

More recently, convolutional neural networks (CNNs) have aroused a heated discussion in the scientific and industrial communities [28]. Qin reported a relation classification task utilizing a CNN approach to automatically control feature learning from raw sentences [29]. Yan reported a depth estimation method using two CNN architectures from raw images [30]. Zhu proposed a framework with a fully convolutional network (FCN) and deep CNN for traffic sign detecting and recognizing [31]. In [32], Chen, Z.Q. et al. used 256 signal statistic features to construct a 16 × 16 feature map and then utilize CNN for the gearbox fault identification, and the classification was reported as 98.35% accurate. According to the studies mentioned above, the CNN received better results in comparison with the peer method.

**Figure 2:**
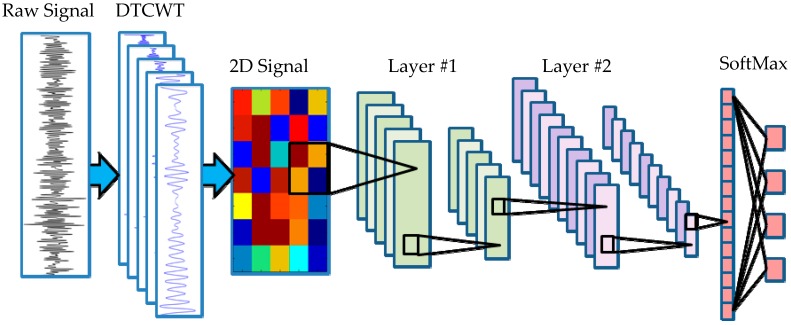
Schematic of the propose method in this paper. DTCWT, dual tree complex wavelet transform.

Inspired by the idea of CNN, we present an intelligent fault diagnosis method using wavelet enhanced CNN. The schematic of the proposed method is shown in Figure 2. In this method, DTCWT is employed to acquire the multiscale signal features with a fixed decomposition level from the raw vibration signal. The CNN approach is utilized to automatically enable fault feature recognition from the multiscale signal’s features. After the network weight coefficients are set via a training set (labeled data), the novel method is more efficient for fault recognition compared with traditional methods, and also makes mechanical fault diagnosis move toward real artificial intelligence.

The contributions of this paper are summarized as follows.

(1) The paper proposes an intelligent fault diagnosis method, which combines the traditional decomposition signal analysis technology and artificial intelligence technology. Different level DTCWT decomposition signals comprise a component matrix of multiscale signal features. Then, CNN is employed for fault pattern recognition. Because of the engagement of CNN to learn the features, the model does not depend on any prior knowledge.
(2) A gear fault case study is used to verify the proposed method. The experimental result shows that the proposed method has good generalization ability for fresh signals.

The rest of this paper is organized as follows. The signal decomposition method DTCWT is briefly described in Section 2. The learning method CNN is presented in Section 3. Section 4 gives the proposed fault diagnosis method. Section 5 details a simulation experiment for the fault classification based on the CNN. A typical gear fault is carried out in Section 6, and the model training process and a validation experiment are also presented. The major findings of this work are summarized in Section 7.

## 2. Signal Decomposition

The useful transient features are usually buried in heavy background noise and other irrelevant vibrations [33]. A basic challenge of CM-FD is how to properly extract the fault feature under a lower-level signal noise ratio (SNR) [34]. To acquire an acceptable calculation time for the pattern recognition in this paper, proper data pre-processing is necessary. In the literature, DTCWT is reported to enjoy merits such as a higher degree of designing freedom, approximate shift-invariance, and inhibited frequency aliasing [35]. Therefore, compared with conventional waveforms implemented in the time domain, DTCWT has a better extraction ability for the periodic non-stationary fault features. In this research, DTCWT is utilized to perform the multiscale decomposition on the raw acquired data.

### 2.1. DTCWT Framework

The wavelet transform has been exploited with great success across many applications. In wavelet theory, a record of a finite energy signal x(t) can be decomposed in terms of wavelets and scaling functions, shown as below.

(1) $$\begin{array}{ll} x(t)= & \sum_{n=-\infty }^{\infty }c(n)\phi (t-n)\\ & \;+\sum_{j=0}^{\infty }\sum_{n=-\infty }^{\infty }d(j,n)2^{j/2}\psi (2^{j}t-n) \end{array},$$

where ϕ(t) is the scaling function, and ψ(t) is the wavelet function. The scaling coefficients c(n) and wavelet coefficients d(j,n) are computed via the inner products:

(2) $$c(n)=\int_{-\infty }^{\infty }x(t)\phi (t-n)dt,$$

(3) $$d(j,n)=2^{j/2}\int_{-\infty }^{\infty }x(t)\psi (2^{j}t-n)dt,$$

Although wavelet transform has many advantages, there are still some fundamental problems such as fixed oscillatory behavior, shift variance, aliasing, and lack of directionality. Inspirited by a Fourier transform, Complex wavelet transform (CWT) $\psi_{\mathbb{C}}(t)$ is proposed with a complex-valued scaling function and complex-valued wavelet:

(4) $$\psi_{\mathbb{C}}(t)=\psi^{ℜe}(t)+j\psi^{ℑm}(t),$$

**Figure 3:**
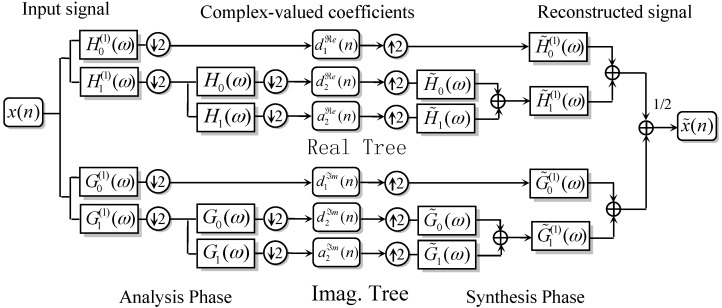
The analysis phase of DTCWT.

The filterbank topology of DTCWT is shown in Figure 3, where the wavelet functions in ‘Tree ℜe’ and in ‘Tree ℑm’ form an approximate Hilbert transform pair:

(5) $$\psi^{ℜe}(t)\approx Hilbert\left[\psi^{ℑm}(t)\right],$$

where Hilbert[·] denotes the Hilbert transform operator.

In the time domain, there is an equivalent expression, as shown in Equation (6).

(6) $$h_{1}^{ℑm}(n)=h_{1}^{ℜe}(n-0.5),$$

where $h_{1}^{ℜe}(n)$ and $h_{1}^{ℑm}(n)$ are real-valued finite impulse response (FIR) filters corresponding to $\psi^{ℜe}(t)$ and $\psi^{ℑm}(t)$. In each filtering tree, the scaling functions of $\psi^{\left(\cdot \right)}(t)$ and $\varphi^{\left(\cdot \right)}(t)$ satisfy the following two-scale relationship:

(7) $$\varphi^{\left(\cdot \right)}(t)=\sqrt{2}\sum_{n\in Z}h_{0}^{\left(\cdot \right)}(n)\varphi^{\left(\cdot \right)}(2t-n),$$

(8) $$\psi^{\left(\cdot \right)}(t)=\sqrt{2}\sum_{n\in Z}h_{1}^{\left(\cdot \right)}(n)\varphi^{\left(\cdot \right)}(2t-n),$$

where the superscript (⋅) can be either ℜe or ℑm. The complex-valued wavelet coefficient series $d_{l}^{\mathbb{C}}(k)$ is calculated via inner product computation between the input signal and the wavelet systems of $\text{\{}\Xi_{j,k}[\psi^{ℜe}]\text{\}}$ and $\text{\{}\Xi_{j,k}[\psi^{ℑm}]\text{\}}$. These complex-valued series are computed using the following expression:

(9) $$\begin{array}{lll} d_{l}^{\mathbb{C}}\left(k\right) & = & \langle x,\;\Xi_{j,k}(\psi^{ℜe})\rangle +j\cdot \langle x,\;\Xi_{j,k}(\psi^{ℑm})\rangle \\ & = & d_{l}^{ℜe}(k)+j\cdot d_{l}^{ℑm}(k) \end{array},$$

where the notation $\Xi_{j,k}[\cdot ]$ denotes the translation and dilation operations simultaneously on a function belonging to $L^{2}(\mathbb{R})$. The mathematical definition of $\Xi_{j,k}[\cdot ]$ is

(10) $$\Xi_{j,k}[\psi ]=\psi_{j,k}(t)=2^{j/2}\psi_{j,k}(2^{j}t-k),$$

where the binary operator 〈⋅, ⋅〉 represents the inner product operation. In the reconstruction phase, $d_{l}(t)$ and $a_{i}(t)$ can be retrieved via

(11) $$d_{l}(t)=2^{\frac{l-1}{2}}\left[\sum_{n}d_{l}^{ℜe}(k)\psi_{h}(2^{l}t-n)+\sum_{m}d_{l}^{ℑm}(k)\psi_{g}(2^{l}t-m)\right]$$

(12) $$a_{J}(t)=2^{\frac{J-1}{2}}\left[\sum_{n}c_{J}^{ℜe}(k)\varphi_{h}(2^{J}t-n)+\sum_{m}c_{J}^{ℑm}(k)\varphi_{g}(2^{J}t-m)\right].$$

Let J be the decomposition stage depth of the dual tree wavelet decomposition in Figure 3, then J+1 wavelet sub-bands, including $\left\{d_{1}(t),\;\ldots ,d_{J}(t)\right\}$ as a detail coefficient series and $c_{1}(t)$ as approximation series, will be produced.

### 2.2. Wavelet Basis Construction

**Figure 4:**
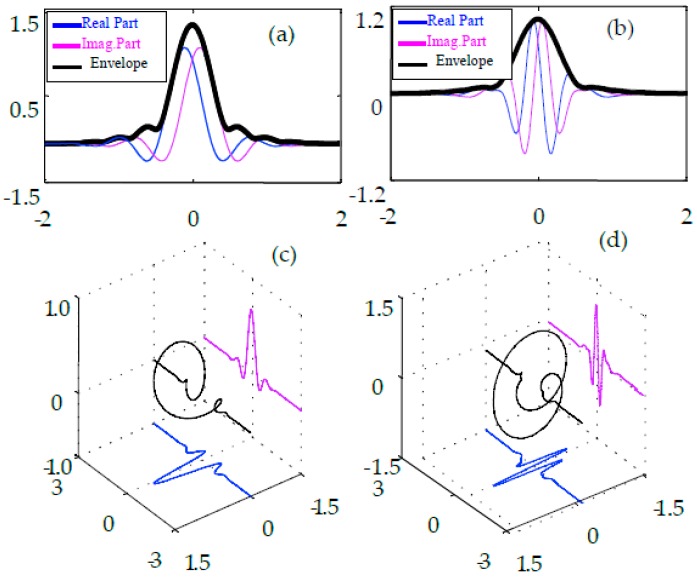
(**a**) Envelope of the complex scaling functions; (**b**) envelope of the complex wavelet functions; (**c**) three-dimensional (3D) plot of the complex wavelet functions and (**d**) 3D plot of the complex wavelet functions.

In this paper, a dual-tree complex wavelet basis, constructed in Ref [16], is employed to acquire the multiscale signal features. The time-frequency atoms of the wavelet basis are shown in Figure 4. As can be observed in Figure 4, this quarter shift basis is advantageous owing to its smooth envelope and annihilated energy leakage.

## 3. Learning Method 

### 3.1. Convolutional Layer

Generally, a CNN is designed to deal with the variability of two-dimensional (2D) shapes. A basic stage in a CNN is composed of a convolutional layer and a pooling layer [36]. Each level consists of a certain number of feature maps, which means that CNNs have a good hierarchical feature representation ability from a lower level to a higher level [37]. Through the propagation of a CNN, the feature map’s size will decrease layer by layer and the extracted features are more global. Related works show that CNNs are also most popular for audio signal processing in view of its efficiency and higher-level information detection ability through a series of lower-level detectors [38,39].

Given a series of time-domain signals x(t), after the DTCWT multiscale decomposition, the signal can be represented as $x_{S}^{}=[x_{S}^{1},x_{S}^{2}\ldots x_{S}^{L}]$, where *S* is the number of the training samples and *L* is the decomposition level. The corresponding network output can be written as $y=[y_{1},y_{2}\ldots y_{S}]$. Each $y_{S}$ means the output class from the finite set of classes. A convolution operation is the feature extraction process [29]. Defining $w_{ji}^{l}$ as the filters with a sliding filter bank and $b_{j}^{l}$ as the bias, the convolutional layer output feature maps can be expressed as

(13) $$g_{j}^{l}=relu\left(\sum_{i=1}^{m}x_{i}^{l-1}*w_{ji}^{l}+b_{j}^{l}\right),$$

where *i* means the *i*-th input feature map, *j* means the *j*-th output feature map, *l* means the *l* layer, $x_{i}^{l-1}$ means the *i*-th input feature map in the (*l*-1)-th layer, and relu(.) means the activation function in the network is rectified linear units (ReLU).

**Figure 5:**
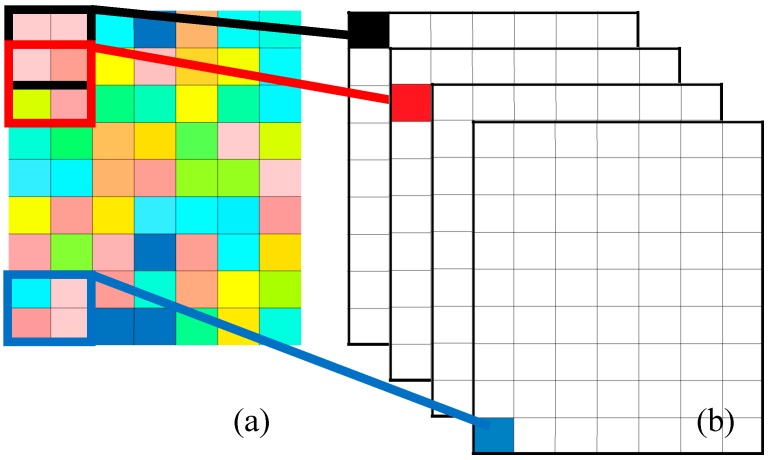
An example of a convolutional layer. (**a**) Multiscale signal and (**b**) output feature maps.

A typical example of a convolutional layer is shown in Figure 5. In Figure 5a, multiscale wavelet sub-bands after DTCWT decomposition are displayed and each column of the colored matrix means the corresponding DTCWT sub-band signal. Each rectangle marked by different colors represents a different convolutional kernel. With the slide of the convolutional kernel, output feature maps are generated (Figure 5b). After the sliding filtering, several feature maps are acquired according to the filter setting.

### 3.2. Pooling Layer

Pooling significantly reduces the computational complexity for the processing steps. Max-pooling and average-pooling are two of the most common pooling methods across various tasks [40]. In this research, max-pooling is chosen for the resolution reduction. Max-pooling can be written as [41]:

(14) $$X_{l}^{j}=down(X_{l-1}^{i}),$$

where down(.) is the sub-sampling function to compute the max value of each *m*×*n* (*m* is the vertical downscale, and *n* is the horizontal downscale) region in the $X_{l-1}^{i}$ map.

### 3.3. Output Layer

The output layer determines the relation label of input signal, and consists of a full-connected layer and a softmax layer [29]. The full connected layer can be presented as

(15) $$a_{j}^{l}=sig\left(\sum_{i=1}^{n}x_{i}^{l-1}\times w_{ji}^{l}+b_{j}^{l}\right),$$

where sig(.) means that the activation function in the network is sigmoid.

The final layer is composed of softmax units. Accordingly, the conditional probability is computed as:

(16) $$p(y_{s}=j|\;a_{s};\theta )=\frac{e^{\theta_{j}^{T}a_{s}}}{\sum_{j=1}^{K}e^{\theta_{j}^{T}a_{s}}},$$

where $y_{s}$ is the actual output of the network, K is the number of the class, $a_{s}$ is the feature vector derived by the full connected layer, and θ is the parameter set to be learned via an algorithm for the first-order gradient-based optimization of stochastic objective functions, Adam [41].

## 4. The Proposed Mechanical Fault Diagnosis Method

**Figure 6:**
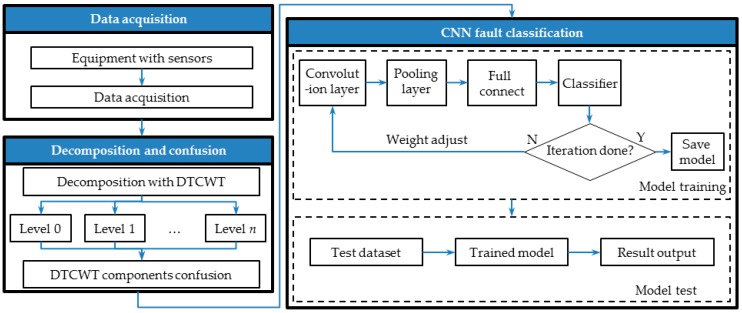
Flow chart of the proposed method. CNN, convolutional neural network.

DTCWT possesses a powerful ability for extracting useful features from vibration signals because of its tight frame and shift invariance [42]. Besides, as a type of feed-forward artificial neural network, CNNs possess a good hierarchical feature representation ability from a lower level to a higher level [32]. Therefore, in this paper, a novel intelligent mechanical fault diagnosis method based on DTCWT and a CNN is proposed to improve the identifying accuracy of mechanical faults. A flow chart of the proposed method is presented in Figure 6, and is illustrated in the following steps.

Step 1: Place the necessary sensors in the measured equipment, and the physical signal can be acquired by a data acquisition system. Meanwhile, the necessary preprocess for the raw signal (anti-aliasing filtering and low pass filtering) is also processed.
Step 2: The acquired signals are decomposed into wavelet sub-bands using DTCWT with a decomposition depth *n*. After that, place the resulting DTCWT wavelet sub-bands as the multiple rows of a matrix, and the DTCWT components are confused into a 2D signal map for the following CNN fault classification. Theoretically, a higher decomposition level will lead to a better result at the cost of higher computational burden. However, in a practice application, computational efficiency is also an indispensable factor. In this paper, the DTCWT decomposition level is set as 7. Therefore, the constructed 2D signal map dimension is 8 × *L*, where *L* denotes the length of the signal.
Step 3: Randomly separate the acquired signal records into two groups, named as the training dataset and testing dataset, and collect an identical number of signal records for each fault type. The training dataset is used to train the CNN framework, which is presented in Figure 2. Due to the limited capacity of the dataset, sixfold cross validation [43] is engaged for the performance evaluation. The proportion of training dataset to testing dataset is 5:1. After the iteration, the model has been saved. The testing dataset is utilized to validate the trained CNN model. In this paper, two convolutional layers are employed for the fault classification in the CNN framework.

## 5. Simulation Experiment

**Figure 7:**
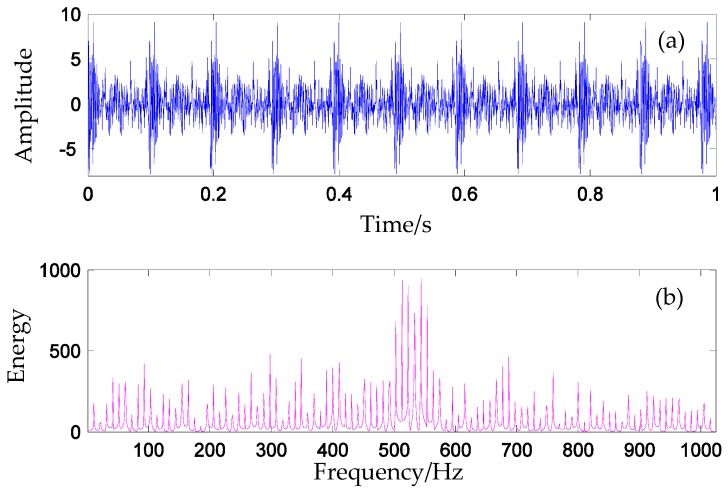
Simulation signal in (**a**) time domain and in (**b**) frequency domain.

The changing health state of gear teeth can lead to variations of amplitude and phase modulations of the meshing vibrations. As such, a trend analysis on the intensity of the modulation components can be effectively used to track the health state of gear pairs [44]. To verify the effectiveness of the proposed method, as well as that of the neural network structure for fault diagnosis applications, simulated gear crack fault signals are established as below.

(17) $$sig_{i}=\sum_{m=1}^{10}Lx_{um}(t)+wgn(t),$$

where $x_{um}(t)=e^{-\beta (t-m\times T_{h})}\sin(2\pi 512t+\phi_{m})$ for 1≤m≤10; *L* denotes the amplitude of the impulse; *wgn*(*t*) is the white Gaussian noise series with 4 dB; the term $a_{um}(t)=e^{-\beta (t-m\times T_{h})}$ represents the periodic amplitude modulation of the *i*-th impulse; and β=90+0.05*(i−1) represents the system’s damping characteristic. The impulse period $T_{h}$ is 0.1025. Meanwhile, random variables $\left\{\phi_{m}|m=1,2,\cdots ,10\right\}$, which are ranged in (−π,π], are utilized to simulate the inconsistency inherent in the periodic impacts due to a variety of factors such as slip, varying load angle, and the transition path of engineering mechanical systems. The sampling frequency is 2048 Hz. In this simulation experiment, 10 simulation signals are constructed for the classification. One of the time domain signals is shown in Figure 7a, and one of the frequency domain is shown in Figure 7b.

**Figure 8:**
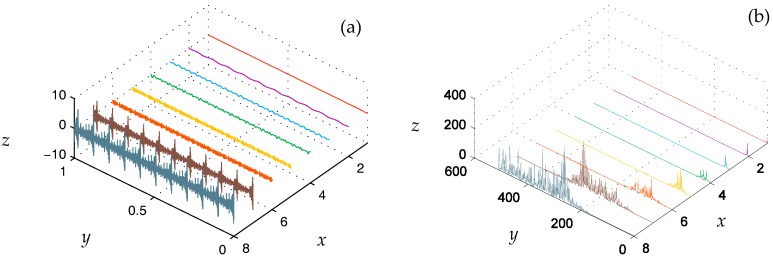
Simulation signal DTCWT component of (**a**) the time domain and (**b**) the frequency domain. Each *x* axis tick in the figure means a DTCWT component where blue grey line indicates the *w*_1_(*t*), the brown line indicates the *w*_2_(*t*), the orange line indicates the *w*_3_(*t*), the yellow line indicates the *w*_4_(*t*), the green line indicates the *w*_5_(*t*), the blue line indicates the *w*_6_(*t*), purple line indicates the *w*_7_(*t*) and the red line indicates the *c*_7_(*t*)*.*

Figure 8a shows the eight signals of the seven-level DTCWT component, where the *x* axis indicates the sub-space signal (1 is the lowest frequency component, 8 is the highest frequency part), the *y* axis is the time axis, and the *z* axis is the signal amplitude. Figure 8b is the corresponding frequency domain of Figure 8a. As can be seen in Figure 8, the main energy of the signal is located in the four relatively high frequency components.

In the vibration measurement, each sampling record contains 2048 discretized sampling points. That is, the duration of each record is 1 second. For each fault class, 1200 records are used for model training and 240 records are used for performance testing. The network used in this simulation experiment is shown in Figure 2.

There are 32 kernels in convolutional Layer #1, and the size of each kernel is set as 3 × 3. Following the convolutional layer, there is an activation Relu layer. After that, there is an additional layer to drop ten percent of the nodes in order to prevent over-fitting. In layer #2, there are 10 feature maps, including convolution, activation, and dropout layers. The configuration of Layer 2 is set similarly to that of Layer 1, except that the kernel size of Layer 2 is chosen as 2 × 2. There is also a full connection layer in the output dimension, which is equal to the fault class number 10. In the output layer, softmax activation is chosen for the classification to represent the categorical distribution, where the Adam optimizer is used to minimize the categorical cross entropy.

**Figure 9:**
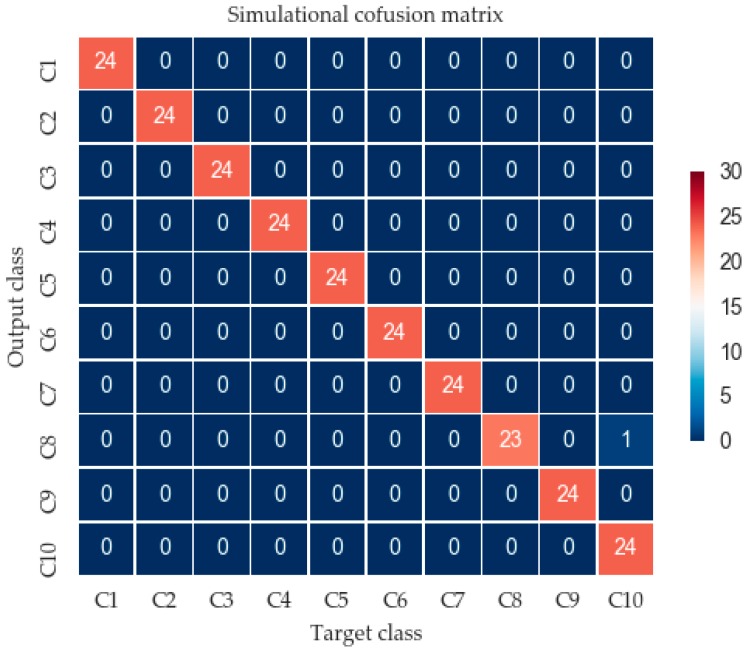
Confusion matrix of the simulation signal.

A confusion matrix is an effective visualization tool to estimate the performance of a classification algorithm [32]. Each column of the confusion matrix represents the instances in a predicted class (output class), while each row represents the instances in an actual class (target class). Figure 9 presented the confusion matrix using the CNN model for 10 patterns, where Ci means the simulated condition in Equation (17).

After 600 epoch iterations, the result shows a great classification effect. As can be seen in Figure 9, the trained CNN model represents a high predicted effect, with a 99.58% accuracy rate and total error of 0.42%.

It is undeniable that the above simulation result shows that the CNN model is of proper fault pattern recognition ability and exhibits good generalization ability. However, the proposed method is only applied to the simulation signal; further actual experiments are also indispensable for its actual performance validation.

## 6. Gear Fault Diagnosis

### 6.1. Experiment and Data Acquisition

**Figure 10:**
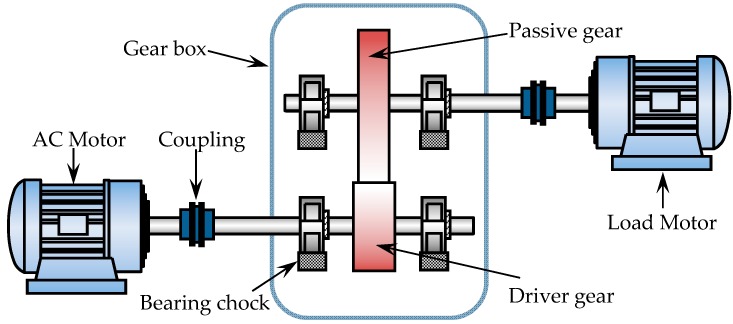
Structure sketch of the test bed.

The data used to train the proposed algorithm in this paper are collected on a custom-built gearbox test rig; the structure sketch of the experimental set-up is shown in Figure 10. The set-up is composed of a speed controller, an alternating current (AC) servo motor, a cylindrical reduction gearbox, a load rotor, balance disk mass, and other auxiliary mechanisms. After starting the set-up, the speed controller is engaged to control the machine such that it works at a constant speed. The load motor is used to provide mechanical loads. The loading force is similar to that of the actual working condition. There is a one stage reduction gearbox in this experiment. The driving gear has 55 teeth, and the driven gear has 75 teeth. The faulty gear is used as the driven gear. Details about the pair of gearboxes are available in Table 1.

**Figure 11:**
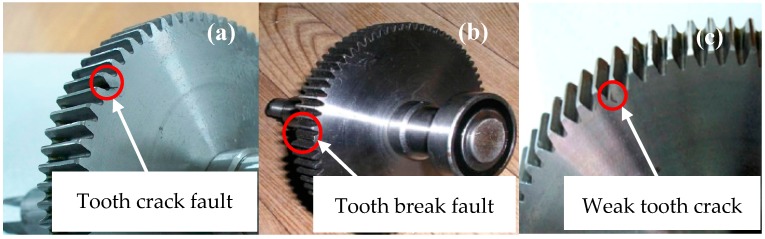
Typical gear faults. (**a**) Tooth crack fault (**b**) Tooth break fault and (**c**) Weak tooth crack fault.

In this research, by removing the driven gear (fault gear), four different fault conditions were researched. Four typical gear faults are simulated in the gearbox test bed: a normal condition tooth crack fault (shown in Figure 11a), a tooth crack fault, a tooth break fault (shown in Figure 11b), and a weak tooth crack fault (shown in Figure 11c). The description of the four conditions of gearbox fault is listed in Table 2.

**Figure 12:**
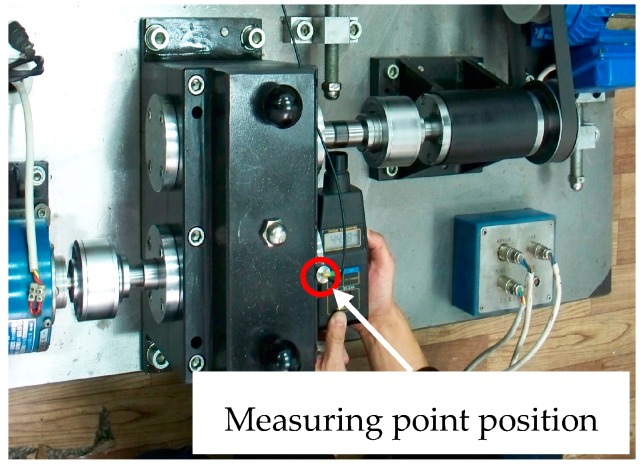
Comprehensive fault diagnosis experimental platform.

The comprehensive fault diagnosis experimental platform is presented in Figure 12. The Sony EX data acquisition system is also employed to acquire the fault signal data. An LC0101T accelerometer is used to collect the fault signal data. As can be seen in Figure 12, the measuring point position is located in the box on the lateral wall of the fault gear. The vibration signals of the gearbox in all operational conditions in Table 2 are measured by the accelerometer and then stored by the data acquisition system, which is equipped with antialias filtering. The sample frequency is set as 12,800 Hz.

**Figure 13:**
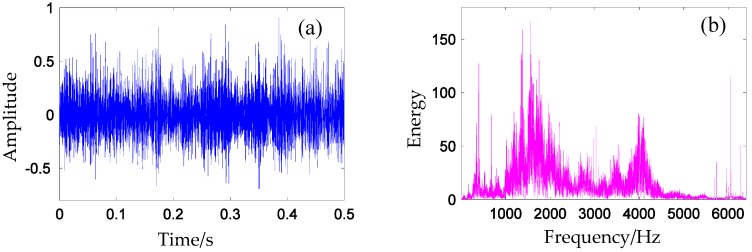
(**a**) Measured vibration signal of test gear and (**b**) Fourier spectrum of the measured signal.

The weak tooth crack fault signal in 0.5 s with sampling rate *fs* = 12,800 Hz (shown in Figure 13) is composed of a periodic sequence of transients occurring with 13 Hz. The current rotating speed is approximately 780 rpm, and the test gear rotating speed is 572 rpm (9.53 Hz). 

**Figure 14:**
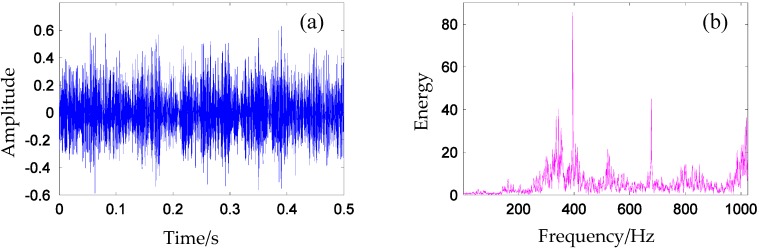
(**a**) Time-domain signal and (**b**) Fourier spectrum of the signal.

As can be seen in Figure 14a, the potential fault modes are masked by noises and irrelevant interference in the time domain vibration signal. Periodic group sparse signals are buried in strong background noise and irrelevant interference. The corresponding Fourier spectrum is shown in Figure 14b. It can be observed from the figure that the energy of the signal is distributed along the whole frequency range. The constituent frequency component is too complicated to identify the characteristic frequency component.

In this research, the fault frequencies are generally lower than 512 Hz, therefore, low-pass filter (1024 Hz) and down sampling operations are used to pre-process the signal so as to enhance the calculation’s efficiency. The time domain signal and the Fourier spectrum after pre-processing are presented in Figure 14.

### 6.2. DTCWT Decomposition and Normalization

**Figure 15:**
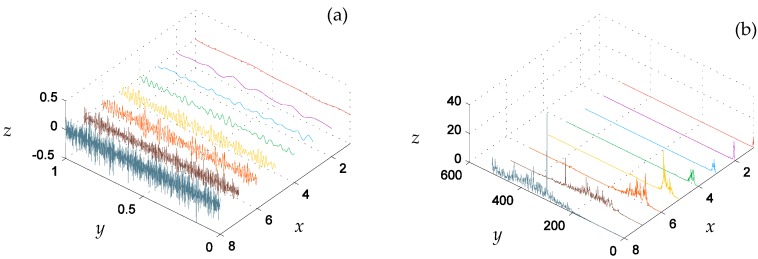
Signal DTCWT component of (**a**) time domain and (**b**) frequency domain. Each *x* axis tick in the figure means a DTCWT component where the blue grey line indicates the *w*_1_(*t*), the brown line indicates the *w*_2_(*t*), the orange line indicates the *w*_3_(*t*), the yellow line indicates the *w*_4_(*t*), the green line indicates the *w*_5_(*t*), the blue line indicates the *w*_6_(*t*), the purple line indicates the *w*_7_(*t*), and the red line indicates the *c*_7_(*t*).

After applying DTCWT to the time domain signal, the decomposition signals of the wavelet sub-spaces and the approximation sub-space are displayed in the zoom-in plots of Figure 15 (1024 points) where the *x* axis indicates the sub-space signal (1 is the lowest frequency component, 8 is the highest frequency part), the *y* axis is the time axis, and the *z* axis is the corresponding physical quantity (amplitude in Figure 15a and energy in Figure 15b). A seven-stage DTCWT decomposition was performed on the acquired signal. As mentioned before, fault features of the signal are submerged in overwhelming interfering contents. Therefore, the fault symptoms are easier to identify from the multiscale signal sub-spaces.

Since the eight decomposition sub-bands, generated by DTCWT, can be considered as a lower dimensional subset in the 2D signal, the one-dimensional (1D) time domain signal can be used to construct a high dimensional signal. As shown in Figure 2, after concatenating the decompositions along the vertical dimension, two dimensional data are constructed. 

Different decomposition sub-bands may be diversified in difference in value owing to different energy distributions. Therefore, adjusting the measured values into uniform scales is necessary. In this paper, the step of feature scaling is used to limit all values within the range [0, 1]. The feature scaling step is defined as

(18) $$X^{\prime }=\frac{X-X_{\min}}{X_{\max}-X_{\min}},$$

where *X* is the original signal, and *X*’ is the new signal after normalization.

### 6.3. CNN Training

The vibration signals were collected from the test rig, mentioned in previous part, under four different operation conditions: (1) normal condition; (2) tooth crack fault; (3) tooth break fault; and (4) weak tooth crack fault. All of the raw vibration signals were collected at a uniform sampling frequency of 12,800 Hz. In the experiment, 630 records of vibration signals were collected for each condition. Therefore, the dataset totally contains 2520 records of signals. Among the 2520 records, 480 records are randomly selected as the testing dataset and the others are used as the training dataset.

The network used in this paper is shown in Figure 2. The input shape of the network for each signal is an normalized patch, which is convolved by a series of two convolutional layers. The size of the kernels in the first layer was chosen to be 3 × 3. Following the first convolutional layer, there is an activation layer to increase the nonlinear properties of the decision function and of the overall network without affecting the receptive fields of the convolution layer. Layer #2 is also a convolutional layer with 10 feature maps. The size of the kernels was chosen as 2 × 2. After a convolutional and a max pooling layer, the high-reasoning in the neural network is done via fully connected layers with full connections to all activations in the previous layer.

After dropping 10 percent of network nodes, there is also a resulting full connection layer. The output dimension of this layer exactly equals to the number of fault types tested in the experiment. In the final output layer, softmax activation is chosen for the classification to represent the categorical distribution.

In this study, we adopt the Adam optimizer [41] to minimize the categorical cross entropy. The cross entropy represents the dissimilarity of the approximated output distribution (after softmax) from the true distribution of labels. Adam is a first-order gradient-based algorithm, designed for the optimization of stochastic objective functions with adaptive weight updates based on lower-order moments.

Batch size and learning rate are two important parameters for the algorithm’s performance. Batch size defines the number of samples that are going to be propagated through the network. Learning rate means how quickly network weights change. Proper parameters can optimize the network training process and reach the best accuracy rate. In this research, different configuration experiments are made to acquire the best performance. All of the experiments in this research were performed under a Linux OS on a machine with CPU Intel Core i5-4460 @ 3.2 GHz. The performance for different configurations of the network's architecture is presented in Table 3. As can be seen in the table, the F score presented an amazing result with 0.9980.

**Figure 16:**
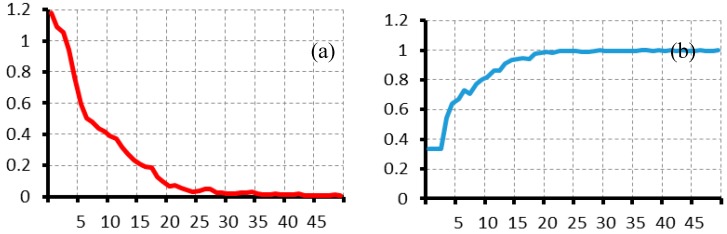
(**a**) Loss function value curves and (**b**) Accuracy rate curves during the training process of the proposed model.

Therefore, we propose to use 60 in batch size, 0.003 in learning rate (red rectangle box in Table 3), and 50 epochs to improve the performance. The performance curves during the training of the established model are shown in Figure 16. The red solid descending curve corresponds to the loss function values for the training sets and for the testing sets during training in Figure 16a. The Figure 16b blue solid ascending curve shows the accuracy rate change during the training process. The results show that the loss function value reaches a stable value after 30 epochs, and the accuracy rate achieves stability after 20 epochs with almost 0.998.

### 6.4. Experiment Results

**Figure 17:**
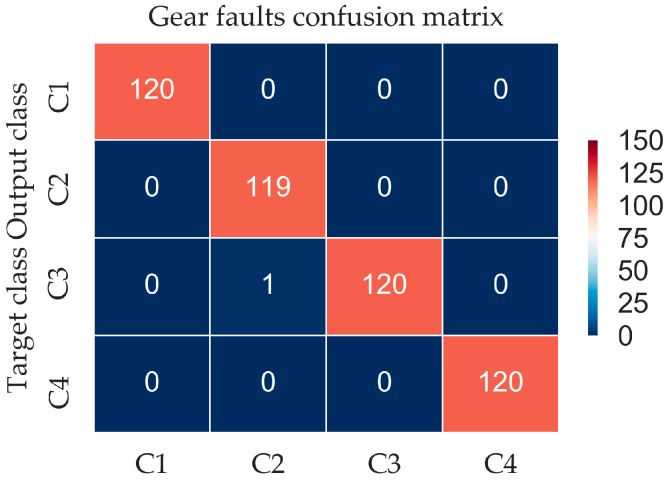
Confusion matrix in gear fault diagnosis.

The confusion matrix of the gear fault diagnosis experiment is shown in Figure 17, where the label meaning of the four conditions of the gearbox can be acquired in Table 2. As can be seen in the figure, the trained model presents a good generalization result, with only one misclassification in the entire 480 testing records. Therefore, the accuracy rate of proposed method is calculated at 99.79%. This result implies that the proposed classification method is not only valid in the simulation signal but also valid in the actual fault diagnosis for gear.

**Figure 18:**
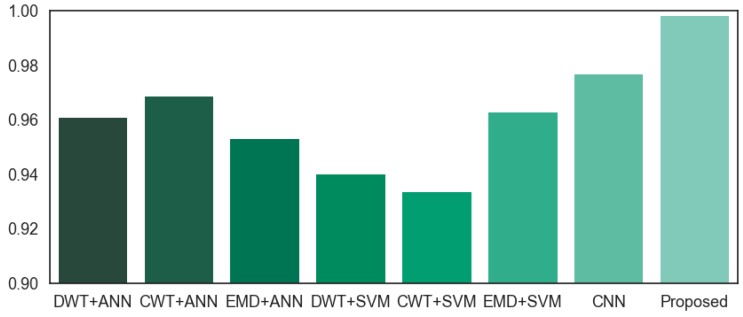
Tested accuracy rate performance comparison.

Performance comparisons among different methods are displayed in Table 4, where the second column is the reported accuracy rates in the corresponding literatures; the last column is the tested accuracy rate for the presented gear fault diagnosis experiment in this research. Figure 18 is the tested accuracy rate performance comparison for the gear fault diagnosis experiment. Compared with the methods mentioned above, the proposed method obtains a higher accuracy, which means that a DTCWT and CNN combination is suitable for gear fault diagnosis.

## 7. Conclusions

In this paper, we propose an intelligent fault diagnosis method using a wavelet enhanced CNN for gear fault pattern recognition, in order to promote recognition accuracy and calculation efficiency. DTCWT is employed to implement multiscale decompositions on gearbox vibration signals. Different wavelet sub-band signals are used to construct the high dimension signals. After normalization, the high dimension signal is used to train and validate the established model. The major findings of this work can be summarized as follows:

(1) A wavelet enhanced CNN is verified to be an effective method to recognize the fault type in mechanical systems. Compared with the traditional CM-FD method, the proposed method is less dependent on prior knowledge as well as excessive artificial diagnosticians.
(2) Different configurations and parameters of the network’s architecture are also studied in this paper (Table 3). Optimized configuration and parameters were identified during the network training process.
(3) The effectiveness of the proposed novel intelligent fault diagnosis method is verified via numerical simulations and a gear fault recognition case study. The results show that the method can distinguish the four types of gear faults with high efficiency.

The proposed diagnosis method for gearbox applications can also be extended to other rotating mechanical systems. In the future, it is worthwhile to investigate its applications to more complicated mechanical fault pattern recognition problems in a completely unsupervised manner. Meanwhile, additional advanced signal processing approaches using some a priori knowledge may enhance its applicability and can enable a more quantitative analysis.

## Figures and Tables

**Table 1:** The parameters of experimental gearbox. AC, alternating current.

Parameter	Module /mm	Tooth Width /mm	Pressure Angle /deg	Number of Active Gear Teeth	Number of Driven Gear Teeth
Value	2	20	20	55	75

**Table 2:** Description of the gearbox operating condition.

Condition	Label
Normal condition	C1
Tooth crack fault	C2
Tooth break fault	C3
Weak tooth crack	C4

**Table 3:** Performance for different configuration (F score).

**BatchSize**	**Learning Rate**					
	**-**	**0.001**	**0.002**	**0.003**	**0.004**	**0.005**
	**20**	0.9980	0.9922	0.9980	0.9980	0
	**30**	0.9980	0.9839	0.9892	0.9754	0
	**40**	0.9960	0.9980	0.9840	0.9922	0
	**50**	0.9951	0.9607	0.9852	0.9833	0.9961
	**60**	0.9951	0.9789	0.9980	0.9804	0
	**70**	0.9922	0.9961	0.9961	0	0
	**80**	0.9931	0.9922	0.9941	0	0
	**90**	0.9794	0.9707	0.9941	0.9902	0.9961

**Table 4:** Performance comparison with different method.

Method	Reported Accuracy Rate	Tested Accuracy Rate
DWT+ANN [22]	95%	96.08
CWT+ANN [23]	98.28%	96.86%
EMD+ANN [24]	93%	95.29%
DWT+SVM [25]	94.33%	94%
CWT+SVM [26]	95.76%	93.33%
EMD+SVM [27]	95.33%	96.27%
CNN [32]	98.35%	97.65%
Proposed method	-	99.79%

## References

[1] Su Z., Tang B., Liu Z., Qin Y. (2015). Multi-fault diagnosis for rotating machinery based on orthogonal supervised linear local tangent space alignment and least square support vector machine. Neurocomputing.

[2] Li Z. (2008). Research on Second Generation Wavelet Theory and Its Application in Fault Diagnosis.

[3] Kia S.H., Henao H., Capolino G.-A. (2015). Gear tooth surface damage fault detection using induction machine stator current space vector analysis. IEEE Trans. Ind. Electron..

[4] Barszcz T., Randall R.B. (2009). Application of spectral kurtosis for detection of a tooth crack in the planetary gear of a wind turbine. Mech. Syst. Signal Process..

[5] Sawalhi N., Randall R.B. (2014). Gear parameter identification in a wind turbine gearbox using vibration signals. Mech. Syst. Signal Process..

[6] Lei Y., Jia F., Lin J., Xing S., Ding S.X. (2016). An intelligent fault diagnosis method using unsupervised feature learning towards mechanical big data. IEEE Trans. Ind. Electron..

[7] Cerrada M., Sánchez R.-V., Pacheco F., Cabrera D., Zurita G., Li C. (2016). Hierarchical feature selection based on relative dependency for gear fault diagnosis. Appl. Intell..

[8] Jingwei G., Niaoqin H., Lehua J., Jianyi F. (2011). A new condition monitoring and fault diagnosis method of engine based on spectrometric oil analysis. Proceedings of the 2011 International Conference on Informatics, Cybernetics, and Computer Engineering (ICCE2011).

[9] Bo Z., Yanan Z., Changzheng C. (2016). Acoustic emission detection of fatigue cracks in wind turbine blades based on blind deconvolution separation. Fatigue Fract. Eng. Mater. Struct..

[10] Gao Z., Lin J., Wang X., Xu X. (2017). Bearing Fault Detection Based on Empirical Wavelet Transform and Correlated Kurtosis by Acoustic Emission. Materials.

[11] Younus A.M., Yang B.-S. (2012). Intelligent fault diagnosis of rotating machinery using infrared thermal image. Expert Syst. Appl..

[12] Gan M., Wang C., Zhu C.A. (2015). Fault feature enhancement for rotating machinery based on quality factor analysis and manifold learning. J. Intell. Manuf..

[13] Du Z., Chen X., Zhang H., Yan R. (2015). Sparse feature identification based on union of redundant dictionary for wind turbine gearbox fault diagnosis. IEEE Trans. Ind. Electron..

[14] Chen J., Li Z., Pan J., Chen G., Zi Y., Yuan J., Chen B., He Z. (2016). Wavelet transform based on inner product in fault diagnosis of rotating machinery: A review. Mech. Syst. Signal Process..

[15] Sun W., Chen B., Yao B., Cao X., Feng W. (2017). Complex wavelet enhanced shape from shading transform for estimating surface roughness of milled mechanical components. J. Mech. Sci. Technol..

[16] Chen B., Zhang Z., Zi Y., He Z. (2014). Novel ensemble analytic discrete framelet expansion for machinery fault diagnosis. J. Mech. Eng..

[17] Wang Y., He Z., Zi Y. (2010). Enhancement of signal denoising and multiple fault signatures detecting in rotating machinery using dual-tree complex wavelet transform. Mech. Syst. Signal Process..

[18] Cira F., Arkan M., Gumus B. (2016). Detection of stator winding inter-turn short circuit faults in permanent magnet synchronous motors and automatic classification of fault severity via a pattern recognition system. J. Electr. Eng. Technol..

[19] Pichler K., Lughofer E., Pichler M., Buchegger T., Klement E.P., Huschenbett M. (2016). Fault detection in reciprocating compressor valves under varying load conditions. Mech. Syst. Signal Process..

[20] Serdio F., Lughofer E., Zavoianu A.-C., Pichler K., Pichler M., Buchegger T., Efendic H. (2017). Improved fault detection employing hybrid memetic fuzzy modeling and adaptive filters. Appl. Soft Comput..

[21] Strączkiewicz M., Czop P., Barszcz T. (2016). Supervised and unsupervised learning process in damage classification of rolling element bearings. Diagnostyka.

[22] Saravanan N., Ramachandran K.I. (2010). Incipient gear box fault diagnosis using discrete wavelet transform (DWT) for feature extraction and classification using artificial neural network (ANN). Expert Syst. Appl..

[23] Dasgupta A., Nath S., Das A. (2012). Transmission line fault classification and location using wavelet entropy and neural network. Electr. Power Compon. Syst..

[24] Ali J.B., Fnaiech N., Saidi L., Chebel-Morello B., Fnaiech F. (2015). Application of empirical mode decomposition and artificial neural network for automatic bearing fault diagnosis based on vibration signals. Appl. Acoust..

[25] Xian G.M. (2010). Mechanical failure classification for spherical roller bearing ofhydraulic injection molding machine using DWT-SVM. Expert Syst. Appl..

[26] Chattopadhyay P., Konar P. (2014). Feature extraction using wavelet transform for multi-class fault detection of induction motor. J. Inst. Eng. (India) Ser. B.

[27] Babu N.R., Mohan B.J. (2017). Fault classification in power systems using EMD and SVM. Ain Shams Eng. J..

[28] Zeng X., Liao Y., Li W. (2016). Gearbox fault classification using S-transform and convolutional neural network. Proceedings of the 2016 10th International Conference on Sensing Technology (ICST).

[29] Qin P., Xu W., Guo J. (2016). An empirical convolutional neural network approach for semantic relation classification. Neurocomputing.

[30] Hua Y., Tian H. (2016). Depth estimation with convolutional conditional random field network. Neurocomputing.

[31] Zhu Y., Zhang C., Zhou D., Wang X., Bai X., Liu W. (2016). Traffic sign detection and recognition using fully convolutional network guided proposals. Neurocomputing.

[32] Chen Z.Q., Li C., Sanchez R.V. (2015). Gearbox fault identification and classification with convolutional neural networks. Shock Vib..

[33] He W., Ding Y., Zi Y., Selesnick I.W. (2016). Sparsity-based algorithm for detecting faults in rotating machines. Mech. Syst. Signal Process..

[34] Gao J., Wang R., Hu L., Zhang R. (2016). 1882. A novel manifold learning denoising method on bearing vibration signals. J. Vibroeng..

[35] Chen B., Zhang Z., Zi Y., He Z., Sun C. (2013). Detecting of transient vibration signatures using an improved fast spatial–spectral ensemble kurtosis kurtogram and its applications to mechanical signature analysis of short duration data from rotating machinery. Mech. Syst. Signal Process..

[36] Duan Y., Liu F., Jiao L., Zhao P., Zhang L. (2017). SAR Image segmentation based on convolutional-wavelet neural network and markov random field. Pattern Recognit..

[37] Tan Y., Tang P., Zhou Y., Luo W., Kang Y., Li G. (2016). Photograph aesthetical evaluation and classification with deep convolutional neural networks. Neurocomputing.

[38] Aytar Y., Vondrick C., Torralba A. (2016). Soundnet: Learning sound representations from unlabeled video. Adv. Neural Inform. Process. Syst..

[39] Sainath T.N., Kingsbury B., Saon G., Soltau H., Mohamed A.R., Dahl G., Ramabhadran B. (2015). Deep convolutional neural networks for large-scale speech tasks. Neural Netw..

[40] Han Y., Lee S., Nam J., Lee K. (2016). Sparse feature learning for instrument identification: Effects of sampling and pooling methods. J. Acoust. Soc. Am..

[41] Kingma D., Ba J. Adam: A method for stochastic optimization. Proceddings of the International Conference on Learning Reprresentations.

[42] Qu J., Zhang Z., Gong T. (2016). A novel intelligent method for mechanical fault diagnosis based on dual-tree complex wavelet packet transform and multiple classifier fusion. Neurocomputing.

[43] Bengio Y., Gr Y. (2003). No unbiased estimator of the variance of k-fold cross-validation. J. Mach. Learn. Res..

[44] Soltani Bozchalooi I., Liang M. (2010). Teager energy operator for multi-modulation extraction and its application for gearbox fault detection. Smart Mater. Struct..

